# Carrier Systems for Advanced Drug Delivery: Improving Drug Solubility/Bioavailability and Administration Routes

**DOI:** 10.3390/pharmaceutics16070852

**Published:** 2024-06-25

**Authors:** Sonia Losada-Barreiro, Sumeyye Celik, Zerrin Sezgin-Bayindir, Sofía Bravo-Fernández, Carlos Bravo-Díaz

**Affiliations:** 1 Departamento de Química-Física, Facultade de Química, Universidade de Vigo, 36200 Vigo, Pontevedra, Spain; cbravo@uvigo.es; 2 Department of Pharmaceutical Technology, Faculty of Pharmacy, Ankara University, 06560 Ankara, Turkeyzerrin.sezgin@pharmacy.ankara.edu.tr (Z.S.-B.); 3 Dentistry Department, Primary Health Unit, Galician Health Service (SERGAS), Calle Mourin s/n, 15330 Ortigueira, A Coruña, Spain; sofiafbravo@gmail.com

**Keywords:** drug delivery systems, administration routes, drug solubility, drug bioavailability

## Abstract

The disadvantages of some conventional drugs, including their low bioavailability, poor targeting efficiency, and important side effects, have led to the rational design of drug delivery systems. In particular, the introduction of drug delivery systems is a potential approach to enhance the uptake of therapeutic agents and deliver them at the right time and in the right amount of concentration at the required site, as well as open new strategies for effective illness treatment. In this review, we provide a basic understanding of drug delivery systems with an emphasis on the use of cyclodextrin-, polymer- and surfactant-based delivery systems. These systems are very attractive because they are biocompatible and biodegradable nanomaterials with multifunctional components. We also provide some details on their design considerations and their use in a variety of medical applications by employing several routes of administration.

## 1. Introduction

The rational design of different drug delivery systems is a progressive interdisciplinary field—that depends on pharmaceutics, chemical science, medicine, polymer science, and molecular biology- that addresses finding the best effective formulation to mediate the desired dose of the drug to the desired site of the action (e.g., unhealthy tissues, tumors), maximizing drug therapeutic efficacy and minimizing side effects. Different factors and parameters must be considered in designing drug delivery systems, including biomaterial properties, route of administration, pharmacokinetics, stability enhancement, the ability of the drug to cross biological barriers, and regulatory aspects, among others.

Carrier systems do not change the fundamental pharmacodynamics properties of a drug with poor water solubility and membrane permeability, but they may change/enhance its pharmacokinetic properties (maximum serum concentration and time to reach it, elimination half-life, among others) to impact its pharmacodynamic performance [1,2,3]. Poor aqueous solubility of the drug, low permeability, and presystemic clearance are common issues for poor bioavailability. Potential advanced drug delivery systems over single- and multiple-dose approaches must keep the effective concentration of the drug in plasma within the therapeutic window.

The way in which a drug is administered can have an important effect on its efficiency. Some drugs have optimal doses for which the efficiency is the highest; lower or higher doses than those considered optimal doses may be hazardous or have no therapeutic effect. The carrier systems can be designed to break down slowly, be selectively delivered to their desired target, and respond to stimuli (e.g., pH or temperature).

## 2. Role of Some Nanocarriers in Drug Delivery

Formulating a new drug molecule is costly and tedious, and thus, maximizing the safety-efficiency ratio of different drugs is the impulse to develop different drug-delivery frameworks, particularly in chemotherapy. They should overcome downsides such as poor bioavailability, toxic effects, and low effectiveness.

**Figure 1:**
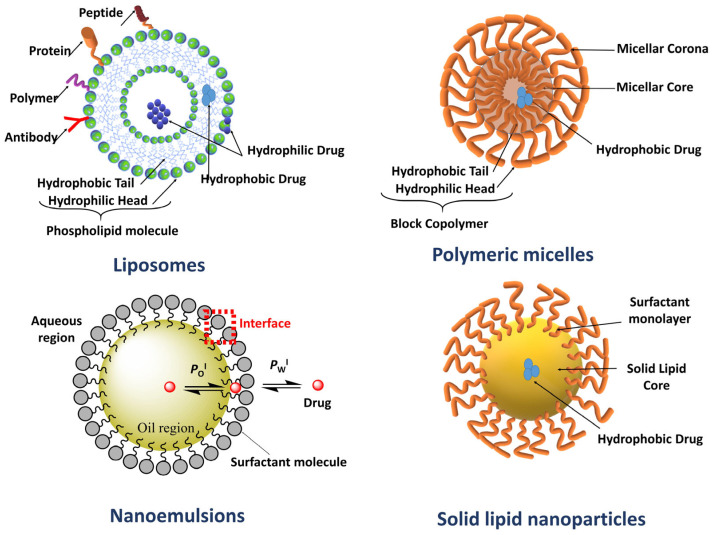
Examples of surfactant-based encapsulation systems. Figures are pictorial, as all colloidal structures are dynamic and have rough surfaces. Adapted from [5].

There are different strategies for encapsulating drugs, as shown in Figure 1, and the selection of adequate delivery systems is key for their potential applications. Recent drug delivery systems provide more advantages than those observed for drugs that are administered conventionally in the form of tablets, pills, suppositories, or injectables, such as improved efficiency, performance, automation, and precision [4]. They are made of nanomaterials with multifunctional components that are biocompatible and biodegradable. Among them, the lipid-, polymer- or cyclodextrin-based nanocarriers are mostly used due to they are biocompatible, have low preparation and modification costs, and are easy to sell up. They have been widely explored for therapeutic and diagnostic approaches because they may enhance the (1) bioavailability and selectivity of the drug, (2) control and target drug release, and (3) pharmacokinetics of the drug. As a result, they may play a key role in disease management and treatment. Lipid-based delivery systems are an umbrella term for several delivery systems, including emulsions, micelles, self-emulsifying drug delivery systems, self-microemulsifying drug delivery systems composed of oils, surfactants, and cosolvents solubilizing lipophilic drug at high concentrations. Polymeric delivery systems include dendrimers, polymer micelles, etc. Some drug delivery systems such as Caelyx^®^, Doxil^®^, Transdrug^®^, and Abraxane^®^ are already commercially available for cancer treatment.

### 2.1. Cyclodextrin-Based Delivery Systems

**Figure 2:**
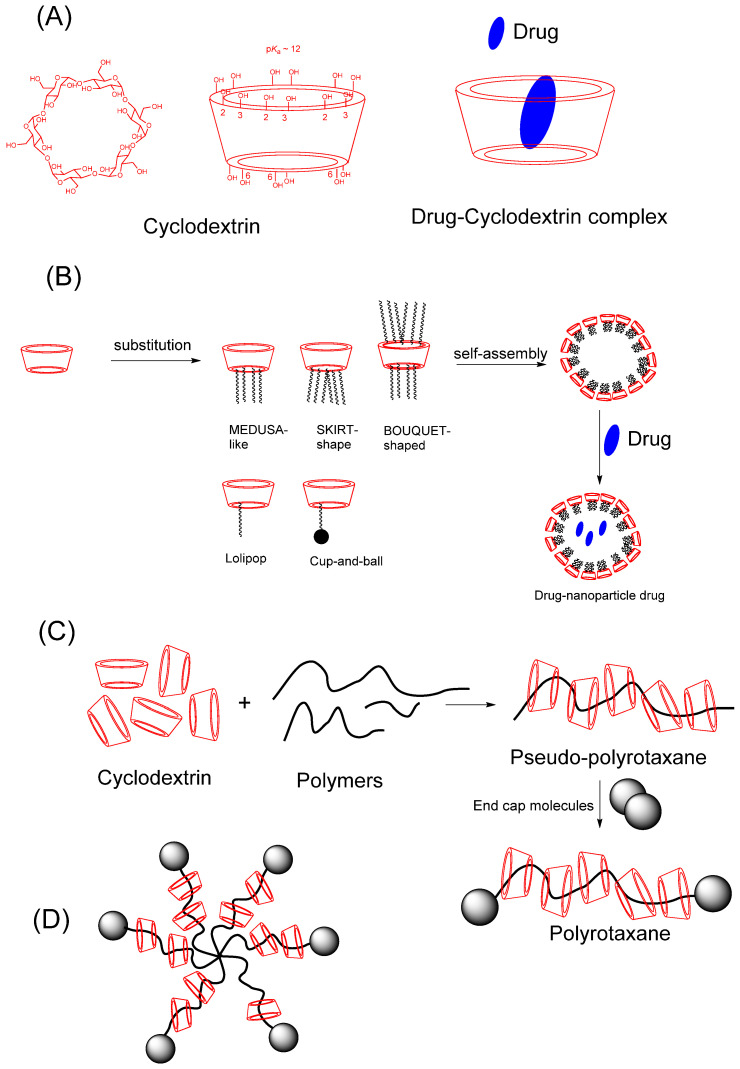
(**A**) Chemical structure of the CDs and the representative spatial conformation with the location of primary and secondary -OH groups [6]; (**B**) Amphiphilic cyclodextrins- monosubstituted and polysubstituted cyclodextrins (structure, self-assembling and drug encapsulation); (**C**) schematic representation of the synthesis of the polyrotaxane supramolecular assembly [13] (**D**) schematic structure *star*-shaped polyrotaxane [7].

Cyclodextrins (CDs) are cyclic oligosaccharides composed of glucopyranose units (Figure 2A), which form a conical cylinder with a hydrophobic inner cavity and a hydrophilic outer surface.

This structure allows them to form inclusion complexes (D-CD) with hydrophobic drugs (D), Equation (1), where *_Kin_*_c_ isthe inclusion constant.

(1) $$Drug(D)+CD⇌Drug(D)-CDK_{inc}=\frac{\left[D-CD\right]}{\left[D\right]\left[CD\right]}$$

The α-, β-, and γ-cyclodextrins with 6, 7, and 8 units, respectively, are widely known and employed as excipients in pharmaceutical formulations. Cyclodextrin rings can also be chemically modified, attached with substituents, and employed to build up large structures, Figure 2B. Hydrophobic chains can be grafted on the primary and/or secondary face of the cyclodextrin rings. These cyclodextrins can self-associate into water-soluble aggregates such as micelles or insert in lipid membranes, improving cell targeting. Polysubstituted amphiphilic cyclodextrins in the primary or secondary hydroxyl groups can give different types, such as medusa-like (substitution of the primary side with thio-, sulfo-, alkyl, amido- or amino- chains), skirt-shaped (modified on the secondary hydroxyl groups will alkyl chains through an ester group), and bouquet-like (modified with alkyl chains on both sides). Other types of amphiphilic cyclodextrins are monosubstituted cyclodextrins giving groups such as “Lollipop” (one alkyl chain on the primary side) or “Cup and Ball” (contain a bulky boc–amino protective group at the end of the alkyl chain). Amphiphilic cyclodextrins can lead to self-assembled nanoparticles, a property that can enhance cell targeting and drug release [6].

It can also find promising new derivatives in development, such as CD-based polyrotaxanes showing improved cellular uptake properties [7]. Polyrotaxanes are derivatives that are constituted by polymer chains and threaded ring-shaped molecules such as CDs. The polymer chains are usually from polymers such as poly(ethylene glycol) (PEG) and poly(-propylene glycol) (PPG). The CDs are threaded onto the PEG chain in an aqueous solution, and end groups are included to avoid the coming out of CDs, Figure 2C. The complexation of CDs with polymer occurs primarily through van der Waals interactions as well as intermolecular hydrogen bonds of CDs. The biomaterial applications of these types of derivatives are increasing due to the possibility of modifying different parameters, including the nature of the polymer, the number of CDs per polymer chain, changes in the CD with functional groups, and/or the addition of side chains to the CDs. Several examples of pharmaceutical applications of polyrotaxane can be found [7], such as pseudopolyrotaxanes of PEGylated insulin with α-CD and δ-CD, which show ~a 4-fold higher hypoglycemic effect [8]. Polyrotaxanes can also be promising candidates for the treatment of Niemann-Pick type C disease, a metabolic disorder producing cholesterol accumulation. Disulfide stoppered Pluronic-based hydroxyethyl-β-CD polyrotaxane was shown to have 100-fold higher cholesterol removal from cells compared to unthreaded β-CD derivatives [9]. Among other CD-polymer conjugates, the star-shaped conjugates with CD core and polymer arms are receiving a particular interest for drug delivery, Figure 2D. CDs are employed as macroinitiators for the synthesis of these derivatives, as well as controlled radical polymerizations such as reversible addition-fragmentation polymerization or atom transfer radical polymerization [7]. For example, β-cyclodextrin-based star polymer with poly(lactic acid)-b-PEG decoration showed high encapsulation efficiency for doxorubicin [7]. Star-shaped CD-based polymers can also be synthesized by conjugating the synthesized polymers to the CD. PEG-b-poly(N-isopropyl acrylamide) grafted β-CD is an example of these derivatives and shows potential interest due to triggered cellular response and tunable self-aggregation [10].

Other interesting CD-based polymeric materials are, for example, chitosan-CD derivatives, combining two biobased oligo/polysaccharides, one of them with the complexing capacity of hydrophobic molecules and another with a cationic chemical structure. Newly, carboxymethyl-β-cyclodextrin grafted carboxymethyl chitosan hydrogel was employed for oral insulin delivery [11].

In this scenario, CDs can act as a crosslinker and a host to encapsulate molecules: the cavity of CDs can carry different types of drugs by forming an inclusion complex, and their hydroxyl groups can be changed to link and add additional cargo. Therefore, the application of CDs has gained wide attraction, increasing the number of clinical trials as it provides stability and sustained release of the guest drug and protects it from external influence and degradation [12].

Improving the Aqueous Solubility of Drugs

CDs play an important role in modifying the physicochemical properties of drugs by improving their apparent solubility through the formation of an inclusion complex D-CD. Table 1 shows different applications of CDs as hydrophilic carriers for drugs with low aqueous solubility. Reported studies show that the aqueous solubility of different drugs (D) increases with the formation of the D-CD complex [14]. Among others, Bozkir et al. [15] formulated an ophthalmic solution (eye drops) employing the formed inclusion complex of chloramphenicol with SBE-β-CD, increasing the solubility of the drug by ~three times at pH 5.5 and by ~two times at pH 7.4. Celtiofur acid was employed in combination with HP-β-CD (1:1 molar ratio), sodium alginate, and poloxamer 407 and 188, improving the solubility of the drug from 0.03 to 2.18 mg/mL [16].

Improvement of the Bioavailability of Drugs

**Figure 3:**
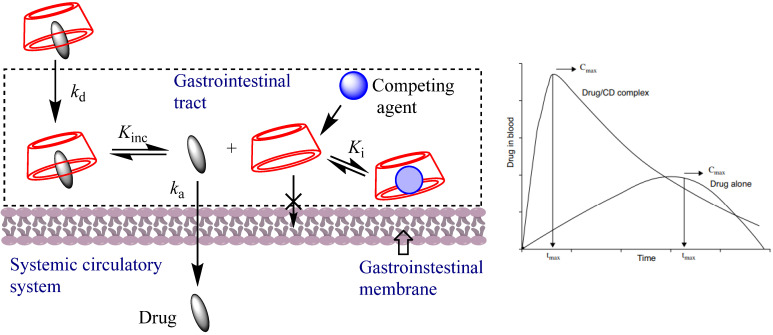
(**Left**) Representative process to release a lipophilic drug. *k*_d_ *K*_inc_, *K*_i,_ and *k*_a_ stand for the dissolution rate constant, the inclusion constant of the complex of the CD with the drug, the inclusion constant of the complex of the CD with the competing agent, and the absorption rate constant, respectively. (**Right**) Enhancement in the bioavailability of a drug by an inclusion complex formation.

**Figure 4:**
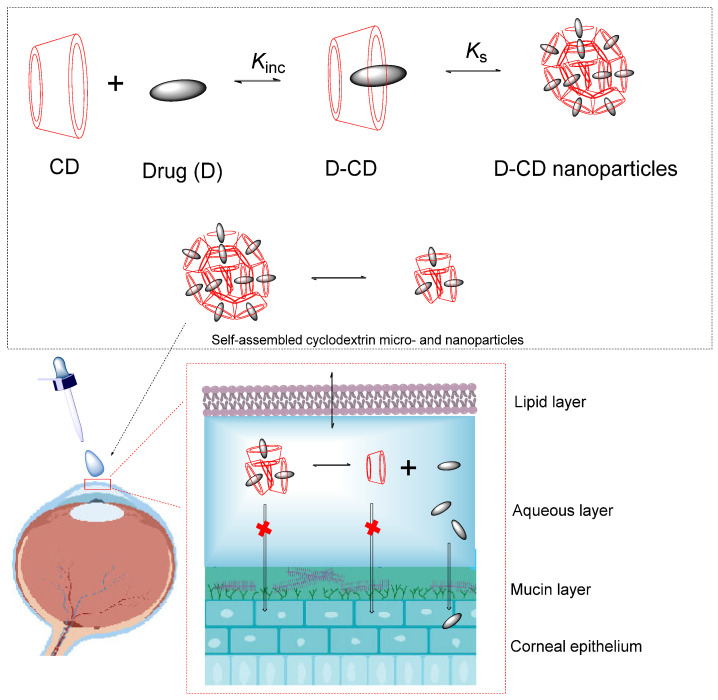
Representative dynamic equilibrium of self-assembled cyclodextrin nanoparticles and drug permeation into the ocular surface by the concentration gradient. *K*_inc_ and *K*_S_ stand for the inclusion constant for the drug-cyclodextrin (D-CD) complex and for the equilibrium constant for the self-assembled cyclodextrin nanoparticles. Adapted from [19].

The bioavailability of the drug can be enhanced by CD complex formation. This is attained by enhancing the drug available at the surface of the biological barrier (e.g., mucosa, eye cornea, or skin), where it partitions into the membrane without disturbing the lipids contained in the barrier. In general, the bioavailability of active drugs depends on parameters such as their solubility, intestinal absorption rate, and dissolution rate. The formation of an inclusion complex improves the dissolution rate, and solubility in gastrointestinal fluids, then increases the amount of drug in blood. On the other hand, the time needed to dissolve the drug from solid form to gastrointestinal fluids and diffusion to blood circulation is reduced, Figure 3. Different mechanisms have been identified for enhancing the bioavailability of active drugs (Figure 3), including (1) improving drug solubility and dissolution rate, (2) preventing degradation of chemically unstable drugs in the gastrointestinal tract, (3) improving permeation of proteins and peptide through the nasal and rectal mucosa by modifying membrane fluidity; (4) compounds such as cholesterol, lipids, bile acid, etc. may act as competitive guest molecule to form an inclusion complex with CD, improving drug release. Dexamethasone eye-drop formulation containing δ-CD nanoparticles is an example, as the presence of cyclodextrins increases the bioavailability of the active drug on the corneal surface, exhibiting a higher concentration and more duration of the drug in the tear firm compared with Maridex^®^ (alcoholic suspension of 0.1% dexamethasone), Figure 4 [19].

Improvement of the Stability and Safety of Drugs

CDs have a key role in the chemical stability of active drugs, retarding or accelerating different types of reactions such as hydrolysis, isomerization, dehydration, and oxidation. For an inclusion complex of stoichiometry 1:1 (D-CD complex, Equation (2) where *k*_d(D)_ and *k*_d(D-CD)_ are the observed rate constant for the decomposition of free drug and of the D-CD complex, respectively). In a variety of reported studies, *k*_d(D-CD)_ < *k*_d(D)_ and the formation of D-CD complexes improve the stability of the drug [20]. Note that the phase-solubility studies are addressed in drug-saturated media, i.e., in non-ideal conditions, and the presence of other excipients, such as buffer salts, polymers, and preservatives, can change the complexation efficiency. Therefore, the complexation media during the formulation of the drug should be similar to the composition of the final formulation.



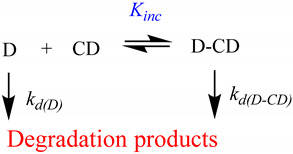

(2)


The stabilizing ability of CDs depends not only on the fraction of the drug that resides within the complex but also on the rate of degradation within the complex (i.e., *k*_d(D-CD)_ value). The stabilization degree will be higher for drugs with high *K*_1:1_ and small values compared with *k*_d(D)_. For example, the hydrolysis of methyl salicylate within HP-β-CD decreases by ~4.2-fold if it is compared to the free drug (*k*_d(D)_ = 4.6 × 10^−3^ min^−1^ and *k*_d(D-CD)_ = 1.1 × 10^−3^ min^−1^) [21]. Another example, the dermocorticoid tixoxortol 17-butyrate 21-propionate, was preserved through the formation of an inclusion complex with β-CD during 30 days at T = 40 °C [22]. Another case is that of beta-lactam antibiotics (e.g., penicillins, carbapenems, monobactams, and cephalosporins), which are prone to degradation reactions due to amide bond hydrolysis and the formation of complexes with CDs were reported to protect B-lactam ring against hydrolytic degradation under acidic conditions and inhibit their degradation [20,23]. Formation of inclusion complex with CDs also seems to be a potential strategy to improve the chemical stability concerning the tetracycline group due to the presence of labile fragments such as tertiary amine and methyl group.

CDs have also been employed to decrease the irritation initiated by drugs [1]. The formation of a complex may minimize or prevent drug toxicity by enhancing the drug efficiency at lower doses. β-CD complex avoids its direct interaction with biological membranes and then minimizes its side consequences and local irritation, providing its therapeutic benefits [1]. Studies have shown that the presence of β-CD enhances the antiviral efficiency of ganciclovir on human cytomegalic virus clinical strains with low drug toxicity [1].

#### 2.1.1. Cyclodextrins as Carriers of Drugs: Administration Routes

CDs are presented in several injectable formulations due to their accessibility and low price, but their application is also interesting for the promotion of ocular and nasal formulations, in which the required drug amount must be dissolved in a small volume of the aqueous solution.

##### Parenteral Formulation

Parenteral drug products are those administrated by routes such as injection routes (e.g., intramuscular, intravenous, and subcutaneous routes) and inhalational and transdermal routes without involving the gastrointestinal tract. Focus on strategies for designing parenteral formulations, CDs have enormous potential to enhance their preparation with poorly aqueous soluble drugs due to their ability to form aqueous soluble complex D-CD, enhancing the drug solubility, its stability, and bioavailability by increasing its circulation time. This has been conducted to show that the elaboration of cyclodextrin-based formulations for parenteral administration is gaining more and more attention in the field of parenteral formulations.

In spite of only a few CDs (α-CD, HP-β-CD, and SBE-β-CD) are contemplated safe for parenteral administration and approved for use in parenteral formulations by the FDA and the EMA, an important number of parenteral formulations, including these CDs can be found available on the market. Under the parenteral administration route, the D-CD complex promptly mingles with blood plasma, contributing to the fast drug release from the complex through the dilution and/or competitive shift because of the attachment of the drug to the plasma proteins, while uncomplexed CDs are clearly by glomerular filtration. A study reported by Loftsson et al. concluded that the impact of CDs on the pharmacokinetics of 13 drugs in aqueous parenteral solution containing CDs after IV administration to rats, rabbits, dogs, and humans was insignificant [21]. Some examples of D-CD complexes commercially available for IV administration are α-CD/alprostadil (Caverject™), SBE-β-CD/voriconazole (Vfend™), HP-β-CD/itraconazole (Sporanox™) and HP-γ-CD/Tc-99 teoboroxime (Cardiotec™), Table 2. For example, itraconazole is an antifungal drug with low aqueous solubility (~1 ng/mL at pH 7), which was increased when HP-β-CD (40% *w/v*) and polyethylene glycol in an acidic medium (pH 4.5) were included in the marketed formulation (250 mg itraconazole/25 mL glass ampoules). Remdesivir, an antiviral drug indicated for the treatment of the Ebola virus, has been the subject of massive clinical studies because of its helpful action in the treatment of COVID-19. Since it presents a poor aqueous solubility, a marketed formulation containing SBE-β-CD has been developed to improve its apparent aqueous solubility, Velkury^®^. In terms of intramuscular formulations can be found SBE-β-CD/ziprasidone mesylate (Geodon™) and SBE-β-CD/aripiprazole (Abilify™), Table 2 [17].

##### Oral Administration

The oral route is the most common route for drug administration but sometimes is challenging for different drugs because of their low solubility, instability, or extensive metabolism. In this sense, CDs have demonstrated great potential to overcome these limitations since different oral tablets are already marketed [24]. Itraconazole oral solution (Sporanox^®^, 10 mg/mL) was developed employing HP-β-CD, Table 3. It is employed in the treatment of candidosis in immunocompromised patients. Its efficiency was also indicated for the treatment of oropharyngeal candidiasis in HIV-infected pediatric older than 5 year [25]. The inclusion complex of piroxicam (Brexim^TM^), Table 3, is indicated in the treatment of musculoskeletal diseases, leading to a faster drug absorption rate while retaining all the analgesic and anti-inflammatory properties of the free drug. Currently, it is presented as sachets, oral tablets, and effervescent formulations in several countries in Europe and Asia. A study addressed the Nimesulide/β-CD complex introduced Nimedex^®^ (Novartis) in the European market as an oral tablet, showing its higher efficiency compared to free nimesulide, leading to a significant decrease in pain intensity 15 min after oral administration, Table 3 [26]. Another example is the inclusion complex of ethinyl estradiol with β-CD, co-administered with drospirenone, and registered as the CD-based oral formulation Yaz (Bayer, Germany). The complex provides a higher chemical stability of ethinyl estradiol, enhancing its shelf-life. CD complexation can also mask bitter drug taste by inhibiting the binding of the drugs to the taste bud receptors located on the tongue. Examples of such formulations on the market include Zyrtec (Losan Pharma), a cetirizine/β-CD complex indicated for symptomatic alleviation of allergy symptoms, Table 3 [24].

##### Ocular Administration

For the ocular administration route, the drug has to be dissolved in a reduced volume of aqueous solution, preserving the hydrophobic properties to be transferred into the corneal epithelium and stroma into the aqueous humor. CDs provide several advantages for developing specific eye drop formulations: (1) improving the aqueous solubility of drugs without interfering with their ability to transfer the hydrophobic barriers and (2) decreasing irritation to the ocular surface. Ophthalmic irritation is a frequent drawback of ophthalmic drugs. CDs are not transferred to the corneal epithelium, but they can keep the drug in the aqueous solution by forming the inclusion complex, leading to a higher bioavailability at the surface of the corneal barrier. As examples, it can be found two marketed eye drop formulations, including CDs: antibiotic clorocil™ (MB-CD/chloramphenicol) and the anti-inflammatory Voltaren ophthalmic™ (HP-δ-CD/diclofenac sodium IC) under several brand names [27], Table 4. Similarly, cetirizine complexes with α-CD, β-CD, and δ-CD remove intense irritation after ophthalmic administration of the free drug (an antiallergic drug) [28]. Some ophthalmic solutions are aqueous solutions in which the drug is susceptible to chemical degradation. Dipivefrine (sulfobutyl ether β-cyclodextrin) enhances the aqueous stability of dipevifrine by ~15–30 and ~20–200 times at pH 5 and pH 7.4, respectively [28]. Another observed advantage of cyclodextrin-based eye drop formulations is that they can reduce the frequency of drug administration, enhancing ocular acceptance of the drug [26]. Significant improvement in the chemical and enzymatic stability of ganciclovir prodrugs was reported for its combination with HP-β-CD [28].

##### Nasal and Intranasal Administration

Nasal drug administration can be an effective alternative route for systemic, local therapy, and brain targeting of drugs [30].

Approved products such as Baqsimi™, indicated for the treatment of diabetes mellitus, contain β-CD for improving the stability, solubility, and bioavailability of glucagon [31]. Another commercial product is Aerodiol^®^ containing RM-β-CD/17β-estradiol where the CD enhances more than 1000-fold the solubility of the drug, leading to the absorption of therapeutically significant drug doses in a reduced volume fit for nasal administration of the active agent glucagon [27].

Formulations of midazolam containing 12% (*w/w*) of RAMBEB, indicated for sedation previously surgical, diagnostic, or dental actions and for the treatment of seizures in pediatric and adult patients, allow a higher drug dose (30 mg/mL of midazolam) compared with to that prepared employing propylene glycol (27.8 mg/mL) or propylene glycol and PEG 400 as cosolvents (25 mg/mL) [32].

In addition to the pharmaceutical formulations currently on the market, it is worth highlighting the more recent in vitro and ex vivo studies on CD-based nasal and intranasal formulations, leading to higher solubility and permeability of the active drug through the nasal mucosa [30]. For example, it was higher stability and aqueous solubility when the anti-rhinovirus drug Disoxaril was complexed with DM-β-CD, increasing its permeation across excised bovine nasal mucosa [33]. Idebenone/HP-β-CD complexes are indicated for nasal administration since they increase the therapeutic action of idebenone as a neuroprotector. Nasal formulations of midazolam complexed with RM-β-CD enhance the bioavailability of midazolam by around 90% compared to the free drug [34].

### 2.2. Emulsion-Based Delivery Systems

Nanoemulsions have proven to be versatile and effective drug delivery systems due to their variety of uses, including drug delivery, where they serve as effective carriers for bioactives in a variety of administration routes. Their parenteral delivery has been employed to meet nutritional requirements, control drug release, and deliver and target drugs to needed locations.

**Figure 5:**
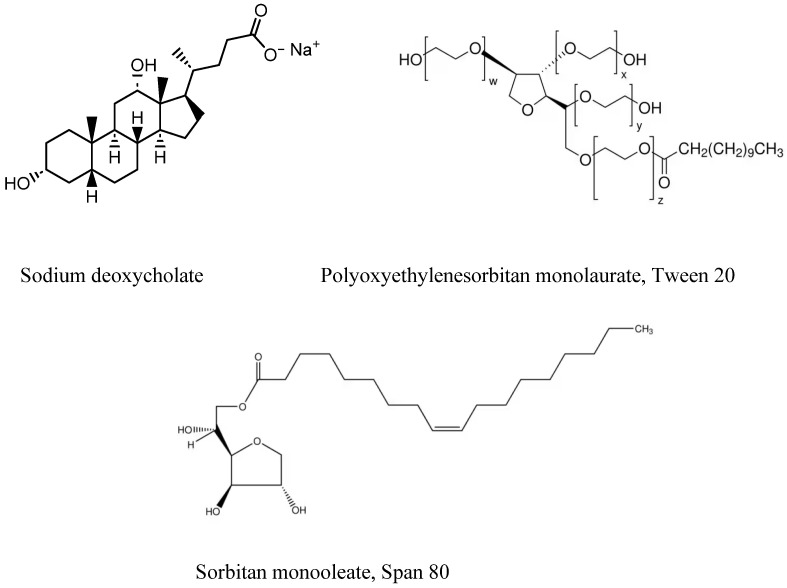
Chemical structure of surfactants that are commonly employed for the preparation of emulsion-based delivery systems.

Nanoemulsions are water-in-oil (W/O) or oil-in-water (O/W) dispersions of two immiscible liquids (e.g., oil and water). These emulsified droplets are kinetically stabilized by adding the appropriate surfactant. The formulation of O/W emulsions is typically a 5–20% oil/lipid phase of several sources such as coconut oil, rice bran oil, sesame oil, soybean oil, etc., often grouped as short chain, medium chain or long chain triglycerides, employed individually or in combination. The surfactants that are commonly employed are polyoxyl 35 castor oil, sodium deoxycholate, solutol HS-15, polyoxyethylene sorbitan monolaurate 20, 40, 60, and 80 (Tweens), sorbitan monolaurate 20, 40, 60 and 80 (Spans) among others, Figure 5.

In contrast to microemulsions [35], nanoemulsions require more energy to be produced (to form a fine emulsion) and are frequently prepared by energy-driven methods.

To guarantee the stability of emulsified systems, it is mandatory to administer them to the body. It depends upon different factors such as the composition of emulsified systems, surface tension, pH, droplet size, electrical charge of the droplets, etc. The higher the zeta potential, the higher the electrostatic repulsion between droplets, enhancing their stability. Droplet size is a critical parameter of the formulation of emulsions because it has a significant influence on the physical stability and on toxicity of emulsions. Emulsion droplets higher than 5 µm can be confined in the lungs and produce pulmonary embolism. The droplet size of an injectable emulsion should be less than 500 nm [36]. Parameters such as the nature of oil and surfactants, the method applied to prepare the emulsified system, pressure, number of cycles, and temperature can be affected by the droplet size of the emulsion.

#### 2.2.1. Effects of Micro- and Nano-Emulsions on Drug Properties in Pharmaceutical Formulations

Micro/nano-emulsions are non-toxic and non-irritant to human and animal cells, which makes them ideal therapeutic agents. Several drug-containing emulsions can be found in the market, and others are under preclinical trials or development. Table 5 displays examples of natural and synthetic drugs with described pharmaceutical action, which can be included in emulsion-based systems.

Micro-/nano-emulsions show different potential advantages as drug delivery systems:

- They can be fabricated easily for the delivery of different drug components with different properties.
- They can have enhanced loading capacities, long-term stability, and improved bioavailability and protection. Studies have reported their broad range of applications to improve the pharmacokinetics and bioavailability of water-insoluble drugs. The dynamics of drugs can also be enhanced by subtly changing the composition of the emulsified systems to modulate their release. For example, several drugs, such as charthromycin, sodium phenobarbital, and all-trans-retinoic acid, among others, were reported to have improved stability in emulsions, probably because of minimizing their oxidation or hydrolysis [37]. Regarding the improved bioavailability, drugs such as Paclitaxel (a drug with proven activity against different tumors) in nanoemulsion formulations showed enhanced bioavailability as it was presented longer time in the systemic circulation compared to that contained in bulk aqueous solution [38].
- They can be applied as liquid formulations, creams, sprays, gels, aerosols, and foams and can be administered by different routes, including oral, intravenous, intranasal, ocular, etc. [39]. When administered orally, the small size of droplets in micro/nanoemulsions and their ability to solubilize poorly aqueous soluble drugs provides a strategy to increase the rate of drug dissolution and subsequently expected bioavailability.
- Some aspects, such as the ability to undergo direct paracellular/transcellular transport and prolonged gastric retention, contribute to enhancing drug bioavailability and minimizing the dose of drugs.
- They can minimize pain, thrombophlebitis, and irritation. For example, the administration of Diazemuls^®^ to 2435 patients reported a reduction in pain, i.e., only 0.4% of patients showed pain, with no blushing of skin compared to the administration of diazepam formulation Valium^®^/Assival^®^ (solution composed of propylene glycol/ethanol/benzyl alcohol) [40]. Likewise, clarithromycin-based emulsion was reported to produce less pain than clarithromycin lactobionate solution [40].
- They can reduce the toxicity of drugs. As an example, the emulsion formulation (1.2% egg phospholipid/10% soybean oil) of cyclosporine minimizes the severe nephrotoxic side consequences connected with cyclosporine in the Cremophor^®^ EL formulation [40].

#### 2.2.2. Micro- and Nano-Emulsions as Carriers of Drugs: Administrations Routes

**Figure 6:**
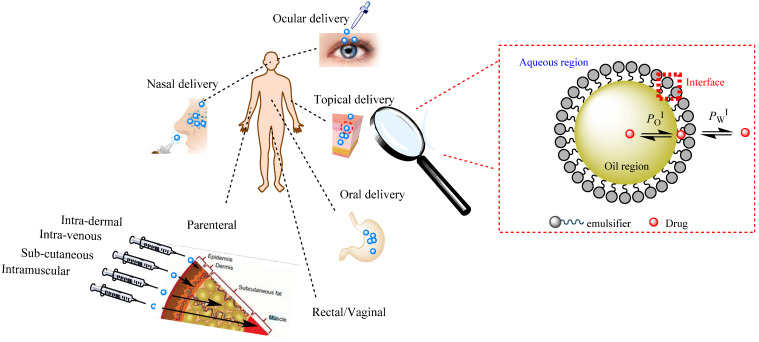
Administration routes for emulsion-based drug delivery systems.

Different routes of delivery, including transdermal, oral, ocular, parenteral, and others, have been the focus of the medical research [35], Figure 6. Surface engineering of micro- and nano-emulsions can help to target the specific sites in some diseases and limit the side effects. Due to their submicron size, they can easily be targeted to the tumor area, acting as carriers of different anticancer drugs, neutron capture therapy agents, photosensitizers, and diagnostic agents. In this sense, the development of magnetic nanoemulsions is gaining attention in cancer therapies. Photosensitizers like Foscan^®^ can be delivered to the subcutaneous tissue of the skin in order to promote hyperthermia and then induce free radical generation in photodynamic therapy [41].

##### Oral Drug Delivery

A key condition for oral delivery is sufficient aqueous solubility of the drug in gastrointestinal conditions to avoid a low bioavailability of orally administered drugs and partial treatment of the disease. Emulsified systems can enhance its aqueous solubility, protect it from the external environment of the gastrointestinal tract, and improve permeation across biological membranes, thereby increasing the drug’s availability at the target site. Emulsion formulations allow the introduction to the market of different poorly soluble drugs intended for oral administrations (Table 6). Neoral^®^ and Norvir^®^ have been formulated as self-emulsifying formulations. Cyclosporine is an effective drug in minimizing organ rejection in patients with kidney, lung, and heart transplants, and it has also been employed in the treatment of autoimmune diseases. However, this drug is poorly absorbed, and only approximately 30% of the drug reaches the systemic circulation [42]. The carriers employed for the oral administration of cyclosporine have involucred from crude emulsions (Sandimmune^®^ composed of alcohol (dehydrated), corn oil, polyoxyethylated glycolysis glycerides as well as gelatin and glycerol for the capsule shell, red iron oxide and titanium dioxide) to microemulsion (Neoral^®^ composed by DL-α-tocopherol, ethanol, hydrogenated castor oil, maize oil and propylene glycol, gelatin, glycerol and propylene glycol, and with the addition of coloring agents such as aluminum chloride, carminic acid, iron oxide black, hydroxypropyl methylcellulose, sodium hydroxide and titanium oxide) [43]. The microemulsion formulation of cyclosporine shows a particular improvement in the pharmacokinetic profile [42]. An oral formulation of an antiviral drug is also marketed for the treatment of HIV infection, including Norvir^®^ (ritonavir), providing an enhancement in the bioavailability of ritonavir of up to 331% compared with hard gelatin capsules [44]. Another example of the emulsified-based formulation is Aptivus^®^, which contains ripranavir, a non-peptidic protease inhibitor, and it is introduced for treatment-experienced patients who show HIV-1 strains with PI resistance-associated mutations.

##### Parenteral Drug Delivery: Injectable Lipid Emulsions

The parenteral route injects the drug directly into the blood pool, avoiding the rate-limiting step of oral delivery, i.e., absorption. A nanosize scale provides important benefits of enhanced brain targeting potential, increasing brain concentration of drugs at the targeted site. Table 7 shows marketed parenteral lipid emulsions for nutrition. Intralipid^®^ (20% soybean oil, 1.2% egg yolk phospholipids, 2.25% glycerine, and water) was approved in Europe in 1962 as the first safe lipid-based nutritional nanoemulsion. These emulsions provide a high content of essential fatty acids such as linoleic and α-linolenic acids and vitamins E and K. However, the high content of ω-6 polyunsaturated fatty acids (ω-6 PUFA ~52–55% in soybean oils) has raised concerns in critically ill patients and patients with compromised immune function. It may increase the formation of arachidonic acid, which enhances the synthesis of pro-inflammatory mediators, among others, and a correlation between immunosuppressive actions and high ω-6 PUFA content was found [40]. To overcome the disadvantages of long-chain triglycerides (LCTs), an emulsion composed of a mixture of soybean oil and medium-chain triglycerides (MCTs, from coconut oil) was developed, Lipofundin^®^ with a 50% less ω-6 PUFA. MCTs do not boost the formation of pro-inflammatory mediators, and their presence has other advantages, such as decreasing their accumulation in adipose tissues and the liver. As a disadvantage, their use may be restricted in patients with diabetes mellitus or other clinical complications intensified by acidosis or ketosis [40]. In order to provide essential fatty acids and minimize levels of medium-chain fatty acids, structured triglycerides-based emulsions were introduced, e.g., ClinOleic^®^, composed of 80% olive oil and 20% soybean oil, has also emerged as an excellent choice for immunocompromised patients, being more tolerable to the liver than MCT/LCT emulsions. The most recently developed parenteral emulsions SMOFlipid^®^ (15% fish oil, Table 7), Lipoplus^®^ (10% fish oil, Table 7), and Omegaven^®^ (pure fish oil emulsions) include in their composition fish oil. Fish oil is enriched in ω-3 polyunsaturated fatty acids such as DHA (docosahexaenoic acid) and EPA (eicosapentaenoic acid), and several studies have reported beneficial properties by inhibiting the formation of the pro-inflammatory cytokines (TNF-α, TL-6, and TL-1β) or by preventing cardiac arrhythmias.

Note that the adverse effects of high lipid content in parenteral nutrition can be diluted when drug delivery systems are employed when considering small-volume injections. As an example, for Intralipid^®^ 20%, 175 g of fat is the daily dosage recommended for an adult (70 kg). The administration of an injectable anesthetic as Diprivan^®^ (10 mg/mL with 10% *w/v* fat), the daily fat is less than 100 g, considering 24-h inflation at a rate of 6 mg^−1^ kg^−1^ h^−1^. Table 8 describes a representative marketed drug containing injectable emulsions.

Most parenteral lipid emulsions also contain an important nutritional component such as vitamin E (tocopherol), an oil-soluble antioxidant that protects the properties of biological membranes by minimizing lipid peroxidation, Table 8. Different isoforms of tocopherol can be differenced depending on the position and number of methyl groups linked to the chromanol ring, α-, β-, γ-, δ-tocopherols. Their biological activity changes considerably [46]. Seed oils such as olive oil, cotton seed, and sunflower seeds are rich sources of α-tocopherol, while soy and corn oil are rich sources of γ-tocopherol. Thus, tocopherol can be found in the composition of most commercial emulsions. In fish oil-based parenteral emulsions (Omegaven, SMOFlipid, and Lipoplus, Table 7), tocopherol is usually added as an antioxidant to inhibit lipid peroxidation, being its content up to 4–5 fold higher than the tocopherol content of soy oil-based emulsions.

##### Intranasal Drug Delivery

The advantage of intranasal is its capacity to deliver drugs to the central nervous system (CNS) by completely passing the brain barrier (BBB) and by employing emulsion-based drug formulations, various neurological and psychiatric disorders such as migraine, depression, Parkinson’s, Alzheimer’s, meningitis, etc. can be treated. In intranasal delivery, drugs need to diffuse across the nasal mucosa in the olfactory region of the nasal cavity, which has direct access to brain areas. In this sense, micro-/nano-emulsions allow the delivery of drugs across nasal mucosa, supporting their protection and faster diffusion. This route is an excellent way for micro-/nano-emulsions, which have excellent aerosolization capacity, and they are indicated for the local treatment of paranasal sinuses, nasal congestion, and infections [47]. However, to date, there are no approved micro-/nano-emulsions for the delivery of drugs, including corticosteroids or antihistamines, to treat these affections. Some examples of micro-/nano-emulsions can be found in the delivery of an influenza vaccine to mucosal immune cells [48].

##### Topical Drug Delivery

The skin is a multilayer organ, and the outer layer, known as the stratum corneum, is composed of the subcutaneous tissues, dermis, and epidermis, and their composition, particularly in the facial and scalp region, includes cholesterol, fatty acids, keratinocytes, and ceramides. Micro-/nano-emulsions can improve the absorption of lipophilic drugs through the respective skin layers. Several studies have been published on micro-/nano-emulsions for topical skin drug delivery (clinical trials NCT00484120, NCT02445716, NCT01966120, NCT02685592, NCT02367547, etc.) [47]. For example, pharmaceutical preparations for the treatment of psoriasis showed higher bioavailability of paclitaxel when this drug was encapsulated by employing a nanoemulsion formulation [49]. More examples are Ameluz^®^, aminolevulinic acid encapsulated in a nanoemulsion that was applied for the treatment of actinic keratosis and basal cell carcinoma. Topicaine^®^, lidocaine encapsulated in a microemulsion-based-gel product, was indicated for the treatment of localized pain relief. Estrasorb^®^, oestradiol encapsulated in a soybean oil-based nanoemulsion, was applied for the treatment of vasomotor symptoms associated with menopause. In the literature can be found several studies focused on the evaluation of micro-/nanoemulsions for topical delivery of non-steroidal anti-inflammatory drugs such as diclofenac, naproxen, piroxicam, ibuprofen, flurbiprofen, meloxicam among others [50].

### 2.3. Liposomes for Oral Drug Delivery

**Figure 7:**
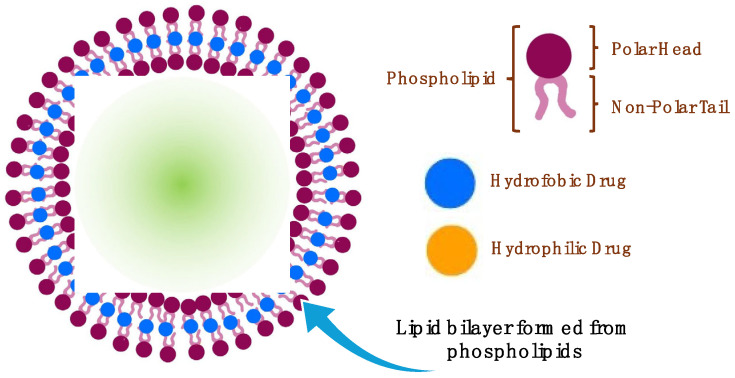
Conventional liposome structure composed of phospholipids.

Liposomes are spherical-shaped closed vesicular systems ranging in size from 50 to 1000 nanometers, which self-close and form a double-layer structure by the hydrophobic/hydrophilic interaction between phospholipids containing hydrophilic and hydrophobic groups in their structure and water molecules (Figure 7) [51]. The physical and chemical properties of liposomes depend on the properties of the phospholipid used and the environment in which it is formed [52]. Liposomes are prepared from natural or synthetic phospholipids and classified according to their size and the number of bilayers in their structure [53]. These nanodrug carriers, which have properties similar to those of the cell membrane, can carry hydrophilic and hydrophobic drugs and can be synthesized easily in large quantities [54]. They have a biocompatible nature and can get degraded in the biological environment without causing toxicity. The external and internal stimuli can trigger drug release from liposomes, and they can simultaneously target the active substance and imaging agent to specific tissues [53]. As a result of the mentioned advantages, it has been shown that liposomes can be administered through different routes and have high potential in oral drug administration.

When administered orally, the stability of liposomes in the gastrointestinal system is the key factor that affects oral bioavailability. Initially, they interact with saliva in the mouth and lead to the emulsification of lipids. Liposomes reaching the stomach from the mouth encounter acidic pH and enzymes. Typically, the pH in the stomach ranges between 1 and 3, but temporary pH elevations of 4 and 5 can be observed due to food intake. The acidic pH may lower the stability of liposomes, and they may undergo morphological changes. At the same time, enzymes such as lipase and protease in the stomach cause liposomes to break down. Liposomes that withstand the harsh stomach environment can pass into the small intestine. The pH value of the small intestine varies between 6 and 7.5 [55]. When liposomal formulations reach the duodenum, some of them are degraded by pancreatic enzymes and bile salts. Bile salts, also known as biosurfactants, can increase the membrane fluidity of liposomes and disrupt their structural integrity. As the concentration and size of bile salts increase, the structural integrity of liposomal formulations deteriorates [55].

The in vivo disposition of liposomes is affected by their particle size and distribution. When the particle size of oral liposomal formulations decreases to nano size, the bioavailability of the encapsulated drug increases as the solubility increases. Large-sized liposomes can be captured by cells of the mononuclear phagocytic system, thus limiting the reach of liposomal formulations into the systemic circulation. To overcome this situation, the PEGylation strategy is used to reduce the elimination of liposomes by mononuclear phagocytic system cells [56,57]. Another factor affecting the bioavailability and stability of oral liposomal formulations is zeta potential. While the uptake of oral liposomes with negative zeta potential by mononuclear phagocytic system cells increases, formulations with positive zeta potential may interact with proteins and cause undesirable effects [58,59]. When developing an oral liposomal formulation, these issues should be taken into consideration, and a suitable design should be made.

#### 2.3.1. Absorption Mechanisms of Liposomes

**Figure 8:**
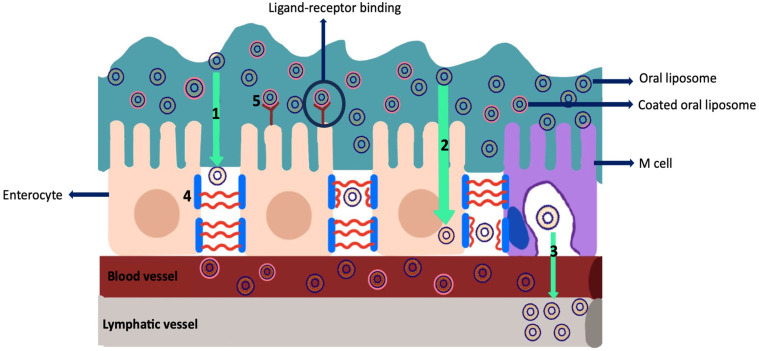
Schematic representation of the passage of oral liposomes from the intestinal environment into the circulation. (1) Paracellular pathway, (2) transcellular pathway, (3) M cell-mediated transcytosis, (4) tight junction, (5) receptor-mediated endocytosis.

Oral liposomal formulations must pass through the intestinal epithelium before entering the systemic circulation. Despite all the disruptive conditions, liposomes can pass through the intestinal epithelium through various absorption mechanisms (Figure 8). Enterocytes lining the small intestine are primarily responsible cells for drug absorption in the gastrointestinal tract. After passing through the mucus layer in the intestine, the drug molecules must pass through the glycocalyx and reach the epithelial layer. Transport through the epithelium occurs via the paracellular or transcellular pathways [59]. The mucus layer in the intestine prevents the passage of oral liposomes through epithelial cells. It is constantly renewed due to the protective effect of the mucus layer against pathogens. For oral liposomes to remain in the intestinal mucus for a longer period, their passage through epithelial cells can be increased via mucoadhesion or immunodiffusion. Various polymers are used to increase the residence time of oral liposomes in mucus. This polymeric coating facilitates the movement of liposomes through the mucus layer and improves their ability to penetrate through the enteric epithelium [60].

Liposomes can be transported through the spaces between neighboring epithelial cells via the paracellular pathway. This transport is controlled by the permeability of the connections between these adjacent epithelial cells and depends on the concentration of Ca^+2^ and Mg^+2^. As the concentration of these cations increases, the permeability decreases. Paracellular transport occurs by passive diffusion and is less sensitive to physiological stimuli. Nanocarriers with sizes smaller than 20 nm tend to be transported paracellularly, and the transport rate can be increased by using absorption enhancers [61,62]. Tight junctions are protein complexes between cells and tightly seal the space between epithelial cells. Tight junctions consist of transmembrane proteins called claudin and occludin. These junctions are transition points that allow or prevent paracellular transport. Tight junction protects epithelial cells against external factors and also prevents the uncontrolled passage of various substances. Tight junction thus provides a selective transition [63]. Molecules can be transported through the epithelial cells via the transcellular route. Transcellular transport occurs through protein-based channels, transporters, and pumps. For the active substance to be transported via the transcellular pathway, it must pass through the cell membrane. This transport is provided by the energy obtained from ATP hydrolysis through the Na-K-ATPase carrier. In transcellular transport, hydrophilic active substances are transported [64,65,66]. Various agents are used to increase the absorption and internalization of the oral formulation. These agents can cross the cell membrane via paracellular and transcellular pathways. For example, ethylenediamine tetra acetic acid provides temporary permeability by causing the tight junction to loosen [59]. By modifying liposomes using ligands, oral bioavailability can be increased by ligand-mediated endocytosis. Intracellular uptake is increased by enabling receptor-mediated endocytosis and accumulation of liposomes in the absorption zone. Receptor-mediated endocytosis is activated by the binding of the ligand to the receptor and enables the internalization of the liposome [67]. Folate receptor is a protein present on the surface of cells, and its expression increases in many diseases. This receptor has an affinity for folic acid. Likewise, the transferrin receptor is a receptor that is abundant in many cells and has an affinity for iron. The cellular uptake of oral liposomes modified with folic acid or iron can be increased via receptor-mediated endocytosis [59]. M cells, a special epithelial cell found in Peyer’s patches, are responsible for the transport of substances to the lymphoid tissues in the intestine. Since M-cells lack mucus secretion, oral liposomes have a prolonged contact time with these cells, which facilitates drug uptake via endocytosis through M cells [68]. Oral liposomes designed for intracellular uptake by M cells can avoid the hepatic first-pass effect if they escape cellular degradation within enterocytes and reach the lymphatic circulation. However, their effect is limited due to their low specificity and low amount, which is approximately 1% [69].

#### 2.3.2. Developed Strategies to Increase the Efficacy of Oral Liposomes

Improving the stability of oral liposomal formulations leads to increased drug bioavailability as they remain in the gastrointestinal tract for a longer time. Within this scope, the lipid composition can be modulated, liposome surfaces can be coated, or the internal phases of liposomes can be thickened [70]. The similarity of oral liposomes to biological membranes is due to their composition of phospholipids and cholesterol. While phospholipids with a phase transition temperature below 37 °C are easily disrupted by bile salts, this effect is less pronounced for phospholipids with a phase transition temperature higher than 67 °C [60]. Bile salts (sodium glycolate, sodium taurocholate, and sodium deoxycholate) can be pre-incorporated into lipid bilayers to prevent the elimination of oral liposomal formulations [71]. This approach provides protection against the destructive effects of bile salts, and such liposomal carriers are called liposomes. In order to increase the stability of oral liposomal formulations, the internal aqueous phases are thickened, increasing the hardness of the liposomes and improving their physicochemical properties. In addition, the surfaces of liposomal formulations are coated with various polymers to protect them from the harsh conditions in the gastrointestinal environment. Due to the presence of this coating, mucoadhesion and mucodiffusion can be increased, and ligand-mediated targeting of epithelial cells is achieved [65]. Table 9 presents examples of strategies developed to increase the effectiveness of oral liposomes.

**Table 9:** Oral liposomal formulations with improved effectiveness.

Formulation Composition	Purpose	Characterization of Optimum Formulation *	Result	Ref.
Insulin Sodium glycolate Soybean phosphotidylcholine Cholesterol	Improving stability	PS: 154 ± 18 nm PDI: 0.340 ± 0.024 EE: 30.2 ± 2.2% BA: 10%	The oral liposomal formulation prepared with sodium glycolate remained stable under harsh conditions. Liposomes were found to be resistant to enzymatic (pepsin, trypsin, and α-chymotrypsin) degradation in vitro.	[72]
Indomethacin Chitosan L-α-distearoyl phosphatidylcholine (DSPC) Dicetylphosphate (DCP) Cholesterol	Increasing mucoadhesion	PS: 300 nm ZP: 37.4 mV BA: 93.1 ± 2.8%	The oral bioavailability of indomethacin was increased with the prepared liposomal formulation. The formulation was retained in the gastrointestinal tract for a long time and a delayed release profile was observed. It has been emphasized that chitosan-coated liposomes can be used for peptide drugs as well as active ingredients with poor absorption properties.	[73]
Doxorubicin Poly(acrylic acid) Poly(allylamine hydrochloride) Egg phosphotidylcholine Cholesterol Stearylamine 7,12-dimethylbenz[α]-anthracene (DMBA)	Ensuring continuous drug release	PS: 520.4 ± 15.0 nm PDI: 0.312 ± 0.062 ZP: +30.4 ± 5.32 mV EE: 63.4 ± 4.26% BA: 29.7%	Due to the fusion of oppositely charged polyelectrolytes in the liposome nucleus, the active substance remained stable in the gastrointestinal system, and its bioavailability was increased. The continuous release feature of the formulation lead continuous exposure of tumor tissue to drug.	[74]
Tacrolimus cyclodextrin complex Pluronic F 127 Egg phosphatidylcholine Paraformaldehyde Coumarin 6	Increasing the solubility of the active ingredient	PS:180.8 ± 8.1 nm PDI: 0.225 ± 0.047 ZP: −4.12 ± 2.43 mV EE: 78.37 ± 9.12%	By forming a complex of tacrolimus, a lipophilic active substance, with cyclodextrin, its penetration into the intestinal mucosa was increased as well as its solubility. The solubility of the tacrolimus cyclodextrin complex coated with pluronic F127 was significantly increased compared to the non-coated formulation.	[75]
Insulin Biotin Soybean phosphatidylcholine Cholesterol 1, 2-distearoyl-sn-glycero-3-phosphatidyl ethanolamine	Improving bioavailability	PS: 150 nm EE: 35–42% BA: 12.09%	High bioavailability has been observed as a result of in vivo studies of the oral liposomal formulation of biotinylated insulin. The bioavailability obtained by biotinylation was found to be twice as high as that of conventional liposomal formulations. As a result of the experiment with Caco-2 cells, it was observed that biotin receptors provide liposomes to be taken into the cells faster. It has been emphasized that the liposomal formulation is taken into the cell through biotin receptor-mediated endocytosis, thus increase the bioavailability. Ex vivo imaging of the GI tracts isolated from rats revealed rapid liposomal absorption after biotin modification (Figure 9).	[76]
Thuricin CD L-α-phosphatidylcholine hydrogenated (soy, HSPC) 1,2-Dipalmitoyl-*sn*-glycero-3-phosphoglycerol sodium salt (DPPG)	Improving stability and bioavailability	PS:103.3 ± 0.7 nm PDI: 0.21 ZP: −46.0 ± 3.8 mV EE: 97.5%	While Thuricin CD undergoes degradation in the gastrointestinal environment, it showed the same activity as free thuricin CD when encapsulated in the anionic liposomal formulation. While its stability and activity were maintained in the gastrointestinal environment, no toxicity has been observed	[77]
Nebivolol hydrochloride Dicetyl phosphate (DCP) Cholesterol (Chol) Pluronic F127 1,2–Dipalmitoyl–sn–glycero–3–phosphocholine (DPPC) Chitosan 1–octadecylamine	Improving bioavailability and increasing the solubility of the active ingredient	Uncoated liposomes PS:146.6 ± 1.6 nm PDI: 0.097 ZP: −14.6 ± 1.6 mV EE: 73.79% Coated liposomes PS: 253.1 ± 5.6 nm PDI: 0.181 ZP: +41.0 ± 2.7 mV EE: 91.72%	The oral bioavailability of Nebivolol hydrochloride in uncoated and coated liposomal formulations was examined. It was found that coated liposomes showed better release properties compared to uncoated ones. At the same time, the formulations had good stability and showed low toxicity in Caco-2 cells.	[78]
Vancomycin conjugate FU002 Egg phosphotidylcholine Cholesterol Glycerylcaldityl Tetraether (GCTE) Cell-Penetrating Peptides -Lipid Conjugate 1,2-Dioleoyl-*Sn*-Glycero-3-Phosphoethanolamine-*N*-(Lissamine Rhodamine B Sulfonyl)	Improving bioavailability	PS:119.6 ± 0.9 nm PDI: 0.10 ± 0.02 ZP: −1.5 ± 0.06 mV EE: 49.8 ± 6.1%	In the in vivo study, the therapeutic efficacy and oral bioavailability of liposomal vancomycin were increased. This study is promising for oral administration of peptide-based antibiotics.	[79]
Vitamin C Soy lecithin Glycerol Sodium ascorbate Sorbitol	Improving stability	PS:81.692 nm PDI: 0.248 ZP: −48.97 ± 1.35 mV EE: 83.479%	When comparing liposomal vitamin C with free vitamin C, stability increased and no leakage of active substance was observed in the formulation. This formulation, produced by new and large-scale production, has increased therapeutic effectiveness with long-term release.	[80]

* PS: Particle Size (nm), PDI: Polydispersity index, ZP: Zeta potential (mV), EE: Encapsulation efficiency (%), BA: Bioavailability.

#### 2.3.3. Clinical Studies Conducted on Oral Liposomes

Along with the extensive research on liposomes, several clinical studies with oral liposomes are also available. In a clinical study, a 1-month pilot study was conducted with an oral liposomal formulation containing 500 and 1000 mg of glutathione per day, which is a critical regulator of oxidative stress. While high glutathione levels were observed in both groups compared to the baseline in glutathione stores, this increase was 440% in the group receiving 500 mg. The effect of oral glutathione liposomal formulation on oxidative stress biomarkers was examined. These biomarkers decreased, especially in the 1000 mg administered group. It was observed that the plasma 8-isoprostane level tended to decrease, especially in the group given 500 mg after 2 weeks. An increase in proliferative capacity and mean lysis time was observed after oral liposomal glutathione administration in both groups. In line with these results, it has been observed that oral liposomal glutathione administration increases glutathione stores and affects the level of oxidative stress [81].

In a randomized study conducted for the treatment of anemia in chronic kidney disease, the effectiveness of intravenous and oral liposomal formulations containing iron was compared. It was observed that the increase in hemoglobin levels in the patient group administered intravenous iron was higher compared to oral liposomal iron. While a significant increase in ferritin levels in the patients was observed in the intravenous iron-administered group from the first month of the study, ferritin levels in the oral formulation remained stable throughout the treatment. The side effect profile was found to be higher in the intravenous group. Although the rate of increase in hemoglobin levels was different, the hemoglobin levels reached similar levels after two applications. As a result of the study, researchers emphasized that oral liposomal iron can be used in the treatment of anemia, but intravenous iron is more suitable in terms of replenishing iron stores and maintaining high hemoglobin levels after discontinuation of the drug, but oral liposomal iron is advantageous due to patient compliance and the observed side effect profile [82].

#### 2.3.4. Patents Related to Oral Liposomal Formulations

There are many oral liposomal and pro-liposomal supplement formulations in the market. The formulations are produced in the form of capsules, syrups, tablets, and sachets. Pediatric usage of liposomal formulations is also possible. Table 10 summarizes the patents obtained for oral liposomal and pro-liposomal formulations. Among the patents received, there are oral liposomal and pro-liposomal formulations that hold promise for carrying drug molecules and food supplements.

Oral liposomes stand out as an innovative approach that has the potential to provide more convenient and effective treatment options for oral drug delivery. Studies show that they have significant potential to optimize the bioavailability of drugs, increasing absorption and minimizing side effects. Formulations should be designed by evaluating critical factors such as liposome size, surface charge, drug release kinetics, and stability. More scientific studies are needed to continue advances in this field and to successfully integrate it into clinical practice.

### 2.4. Polymeric Micelles for Oral Drug Delivery

**Figure 10:**
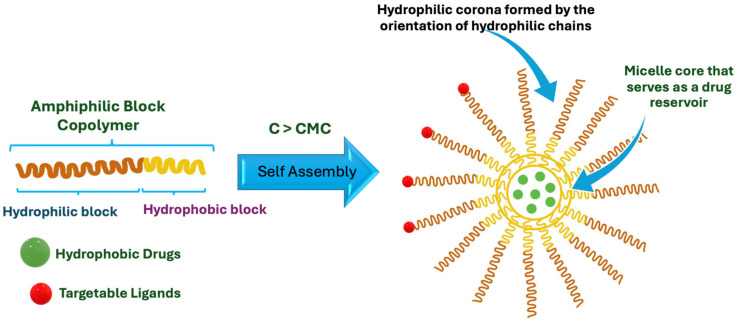
Self-assembly of polymeric micelles from amphiphilic block copolymers above CMC.

**Figure 11:**
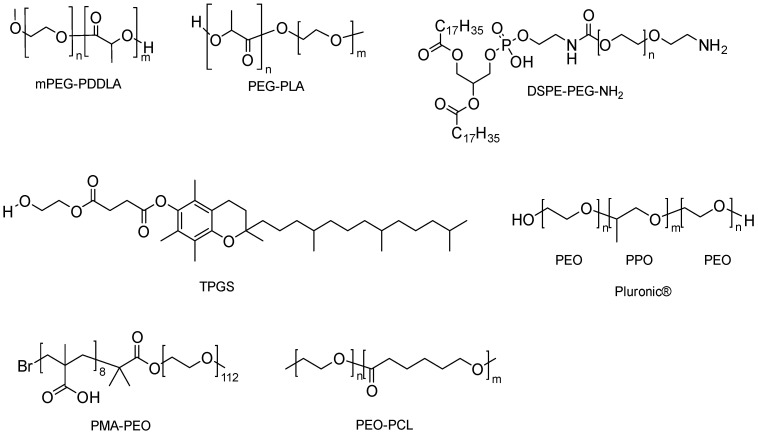
Chemical structure of copolymers used in polymeric micelle preparation.

Amphiphilic copolymers with hydrophobic and hydrophilic parts self-assemble into polymeric micelles (PMs) above their critical micellization concentration (CMC) and act as efficient core/shell drug delivery systems (Table 11). The hydrophobic core of the structure forms a cargo space for hydrophobic drugs, and the hydrophilic corona functions as the steric stabilizer of the entire system (Figure 10) [87,88]. Micellar incorporation of poorly water-soluble drugs improves their solubility and, thus, bioavailability. The potential of solubility enhancement depends on the type and molecular weight of the hydrophobic blocks. Although PEG is generally used as the hydrophilic block in the preparation of PMs, innovative block copolymers are also synthesized using different hydrophobic blocks such as poly(2-oxazoline)s, poly(amino acids), Poly(sarcosine), poly(vinyl pyrrolidone), poly(N,N-dimethylacrylamide), poly[N-(2 -hydroxypropyl)methacrylamide] and poly(methyl methacrylate), Figure 11 [89]. For instance, micelles prepared using high molecular weight poly(ethylene glycol)-block-poly(ε-caprolactone) (PEG-b-PCL) were more effective in solubilization compared to low molecular weight PEG-b-PCL [90]. The therapeutic potential of the drugs can be improved by targeting micelles to specific tissues and shielding healthy tissues from exposure.

Currently, commercial drugs (Genexol-PM, etc.) utilize polymeric micelle technology [91]. Although they hold significant promise in drug delivery via the IC route, their potential for oral drug administration has attracted increasing attention in recent years.

Polymeric micelle formulations can overcome the critical challenges in oral drug delivery (poor aqueous solubility, low stability, low permeability). The nanoscale particle size of polymeric micelles (10–100 nm) facilitates drug transport through the GI mucosa, and the adjustments to their structure enable drug release according to environmental conditions [92]. This can be provided by using different micelle-forming amphiphilic copolymers. Mucoadhesion dominantly affects the drug residence time in the GI absorption site and transmucosal absorption. This can be achieved by designing polymeric micelles to target specific molecules in the mucus layer, such as sialic acid, glycans, glycoproteins, and integrin. Thus, a rational treatment approach can be provided in the treatment of local gastrointestinal system diseases (e.g., Helicobacter pylori).

One of the key advantages of polymeric micelles is their improved stability compared to surfactant micelles due to their low CMC. The kinetic and thermodynamic stability of polymeric micelles highly depends on their CMC value, and as the system drops below the CMC value, the micelles tend to decompose over time [93]. This situation has been interpreted in the literature as PMs being stable upon their dilution in gastric fluids. However, a holistic evaluation is necessary that includes the examination of the system stability against GI enzymes, bile salts, and variable pH.

The fate of orally administered micelles is investigated in several studies [94,95]. The hypothesis of intact micellar uptake through the intestinal barrier and its entrance to the bloodstream was shown at some level. However, further evaluation is still needed to confirm this. He et al. conducted a detailed visualization study using aggregation-caused quenching (ACQ) fluorophores to assess the presence of integral PMs in biological media or tissues [94]. The absorption of integral PMs was limited, and the contribution of the lymphatic transport pathway was low at 1–2% levels. The role of different endocytosis mechanisms on the transport of PMs was also studied, and as an example, clathrin-mediated transport of polyethylene glycol-poly lactic acid (mPEG-PLA) micelles was shown in Caco-2 cells [96]. On the contrary, there are also a few studies that contradict the hypothesis of the absorption of polymeric micelles. Vitamin intestinal absorption was evaluated in bile duct ligated and sham-operated rats after its entrapment in mPEG_5000_-*b*-p (N-(2-hydroxypropyl)-methacrylamide dilactate) based polymeric micelles. The presence of bile salts significantly improved vitamin K absorption, and this was related to the disruption of PMs and absorption of the release of vitamin K via free bile salts [96]. The in vitro drug dissolution release rate from micelles is another important parameter that affects the in vivo performance of PMs after oral administration [97]. PMs that exhibit faster in vitro drug release displayed lower overall absorption and oral bioavailability.

**Table 11:** Several examples of amphiphilic polymers that form polymeric micelles.

Copolymer	Abbreviation/Commercial Name	Reference
Methoxy poly(ethylene glycol)-poly(D,L-lactic acid)	mPEG-PDLLA	[98]
Poly(ethylene glycol)-poly(L-lactic acid)	PEG-PLA	[99]
Poly(ethylene glycol)-b-poly(vinylbenzoxyl)-N,N diethyl nicotinamide	PEG-b-PVBODENA	[100]
Poly(ethylene glycol)-distearoyl phosphatidyl ethanolamine	PEG-DSPE	[101]
Poly(ethylene oxide)–poly(propylene oxide)–poly(ethylene oxide)	Pluronic^®^	[101]
Polyethylene glycol, mono oleoyl glycerol and succinic acid	PEG/MOG/SA	[102]
Poly(ethylene oxide)- block- poly (methacrylate)	PEO-PMA	[103]
Poly(ethylene oxide)-poly(caprolactone)	PEO-PCL	[104]
Linoleic acid-grafted chitosan oligosaccharide	CSO-LA	[105]
Polylactic acid-b-poly(N-(3-aminopropyl) methacrylamide)	PLA-b-PAPMA	[106]
Alginate-graft- N-isopropylacrylamide	Alg-g-PNIPAAm	[107]
Oleic acid grafted low molecular weight carboxymethyl chitosan	OA-CMCS	[108]

Early research on the examination of polymeric micelles’ potential for oral administration was performed on Pluronic and PEG-DSPE copolymers, Table 12. Sezgin et al. revealed the improved retention of polymeric micelles in everted rat intestines. This was interpreted as an advantage for improving the bioavailability of the loaded porphyrin derivate and also the potential of the system for treating localized GI cancers [109]. Further studies agreed with this outcome [110], and the P-glycoprotein (P-gp) efflux pump inhibition with pluronic was considered a reasonable contributor to improved bioavailability [111]. There are several efflux pumps located at the apical surface of enterocytes, and P-gp has received great attention. If the absorbed drug is a P-gp substrate, the drug is actively released back into the intestinal lumen; thus, the oral bioavailability is hindered. In addition to pluronics, the P-gp inhibition role of several other micelle-forming copolymers (Soluplus, TPGS, etc.) was also shown [112,113].

Stimuli-sensitive polymeric micelles were also developed to provide localized drug release via various triggers such as pH, enzyme, etc. [114]. Zhang et al. prepared a micelle-forming amphiphilic copolymer with azo-reductase-sensitive linkage [115]. This copolymer was used to obtain curcumin-loaded polymeric micelles, which additionally contained catechol-modified TPGS (Cat-TPGS) for mucoadhesion. The obtained micelles provided improved drug release through enzymatic stimulation and enhanced local retention. This led to better clinical results in colitis in vivo treatment in mice. Hu et al. synthesized several micelle-forming pH-responsive copolymers (poly(methyl methacrylate-co-methacrylicacid)-b-poly(2-amino ethyl methacrylate) [P(MMA-co-MAA)-b-PAEMA]) that assemble PMs [116]. In the study, PMs entrapped insulin, and the pH-dependent drug release was obtained.

**Table 12:** Recent progress in the literature on oral polymeric micelle formulations.

Drug	Composition	Size	Remarks	Ref.
Cyclosporin A	Polyoxyethylene (10) cetyl ether- hydroxypropyl cellulose	55 ± 1 nm	Caco2 permeability of drug was increased due to the presence of hydroxypropyl cellulose which provides bioadhesion.	[117]
Paclitaxel	D-α-Tocopheryl polyethylene glycol 1000 succinate (TPGS)-modified carboxymethyl chitosan-rhein	193.0 ± 1.0 nm	Absorption of polymeric micelles as a whole was successfully shown in Caco-2 cell uptake studies and biodistribution results on rats. The pharmacokinetic studies also proved the enhancement on the oral bioavailability of the drug.	[118]
siRNA	(CH2R4H2C)-modified methoxy polyethylene glycol-polycaprolactone	50–60 nm	The potential of PMs for delivering siRNA via oral route to treat ulcerative colitis was shown. siRNA stability in acidic environment (pH 1.2 and 6.8) was provided. Additionally, siRNA delivery to the colon and subsequent improvements in clinical symptoms were shown in mice.	[119]
Docetaxel	Oleic acid grafted low molecular weight carboxymethyl chitosan (OA-CMCS)	213.4 ± 9.6 nm	In vitro stability of micelles was shown in simulated gastrointestinal fluids with/without enzymes (pepsin, pancreatin). Improved drug transport through Caco-2 monolayers was related to P-gp inhibiting role of the micelles. The AUC (Area Under the Curve) values increased 2.62-fold via micelles as compared to drug suspension in vivo bioavailability tests.	[108]
Efavirenz	Pluronic^®^ F127 Tetronic^®^ T904	20–27 nm	An 8400-fold improvement in the aqueous solubility of efavirenz was obtained. Oral administration of the micellar efavirenz increased the pharmacokinetic parameters up to 3-fold in in vivo. Slow drug release rate from micelles was pointed as an advantage for improved drug absorption.	[97]
Indomethacin	Soluplus	130 nm	Spray dried powder containing PMs were prepared. The drug solubility was increased and so the ex vivo drug permeability through porcine small intestine in both fasted and fed state conditions.	[120]
Clarithromycin	Thiolated hyaluronic acid-co-oleic acid (CLR-thHA-co-OA), ureido-conjugated thiolated hyaluronic acid-co-oleic acid (CLR-Ur-thHA-co-OA), thiolated hyaluronic acid-co- oleic acid (CLR-PAP-Ur-thHA-co-OA)	210–258 nm	Thiol groups in micellar structure improved mucoadhesion which prolonged the residence time in the GI system that resulted in eradication of *H. pylori*	[121]
Glyburide and vanillic acid	Polyethylene glycol monomethyl ether-block-polycaprolactone (mPEG-b-PCL)	44.6–80.6 nm	The solubility of glyburide approximately doubled via micellar entrapment and a tremendous increase in in vivo bioavailability parameters was observed.	[122]
Paclitaxel	Carboxymethyl chitosan (CMCS) [1-ethyl-3-(3-dimethylaminopropyl)carbodiimide hydrochloride] (EDC) N-hydroxysuccinimide (NHS) Linoleic acid (LA)	94–121 nm	LA-CMCS conjugated micelles were developed to increase the solubility and oral bioavailability of paclitaxel. In in vitro release studies, the formulation showed prolonged release. It was observed that this micellar formulation significantly increased the oral absorption of paclitaxel.	[123]
Mebendazole	Polyvinyl Caprolactam-Polyvinyl Acetate-Polyethylene Glycol Graft Copolymer (Soluplus^®^)	538.7 ± 2.129 nm	An attempt was made to overcome the problem of insufficient solubility of mebendazole with Soluplus. It was found that the prepared formulation was more soluble at acidic pH and fourfold higher bioavailability was obtained.	[124]
Mirtazapine	Solutol (HS15) BrijVR 58 (B58)	298.75 ± 131.87 nm	In the study, it was aimed to increase the solubility and bioavailability of mirtazapine, which has low solubility, by using polymeric micelles. The prepared polymeric micelles were compressed as tablets, and as a result of in vitro tests, it was observed that the relative bioavailability and solubility were increased compared to the market preparation.	[125]

Overall, studies reveal the potential of polymeric micelles to improve the solubility and oral bioavailability of a broad spectrum of drugs, Table 12. In the literature, this improvement is attributed to different underlying mechanisms, and there are contradictive scenarios as well. This reveals that the fate of orally administered micelles should be evaluated case by case since it highly depends on the structure of the micelle-forming polymers, excipients, and the nature of the loaded cargo.

## 3. Conclusions: Challenges Associated with Current Drug Delivery Systems

Drug delivery systems have improved a lot from the common pill with uncontrolled release towards systems with enhanced bioavailability and minimized side effects by influencing the absorption, distribution, metabolism, and elimination (ADME) of an active agent. They play an important role in disease management and treatment. The dose of the active agent is minimized to reduce side effects, but its efficiency and strength remain untouched. This approach is exploited in different lipid-based drug delivery systems such as liposomes, micelles, nanoemulsions, and complexations, among others. For these drug delivery systems, new developments and the introduction of new components have been seen as a dynamically growing development in the last few years. Furthermore, the other advantage of being able to develop new derivatives from biocompatible and biodegradable precursors is the capacity to add different chemical groups to provide cell-specific functions, offering the potential to improve pharmacokinetic properties. Besides, progress on in silico methods is helping to overcome time- and work-consuming trial-and-error in the new approaches and is opening the door for several further applications. Integration of nanotechnology and advanced imaging techniques holds promise for further improving the effectiveness of drug delivery systems.

Target-specific delivery complications are still a challenge in fostering the efficiency of these drug delivery systems and making existing systems available for patient use. One can point out several reasons for the slow incorporation of drug delivery systems to therapeutics: (1) while significant progress has been made in the development of drug delivery systems through the incorporation of different functionalities, many of these approaches fall short of adequately addressing biological barriers, limiting clinical application. For example, the blood-brain barrier (BBB) prevents the entry of carrier particles into the brain and the entire central nervous system, producing ineffectiveness of therapeutic agents in the treatment of cerebral diseases because of the inability to deliver and sustain intended drugs within the brain efficiently; (2) sometimes extrapolation of the behavior from animal models to humans is not easy. Several studies focus on the local administration of small animals, which have significantly different pathologies, physiologies, and immune systems compared to humans. For this reason, just a few of these systems have entered clinical phases; (3) most drug delivery systems are prepared under poorly reproducible conditions, and the methods employed are not standardized. Besides, the developed combinations of polymers, lipids, and other materials are considered ”new excipients,” and thus, antigenicity, clearance, cytotoxicity, and genotoxicity are major issues to be investigated. These are difficulties in large-scale production according to GMP standards, potential toxicity, and premature or burst drug release. In addition, high economic costs and the lack of regulatory standards and guidelines retard the introduction of these systems to the market.

Besides, efforts still must be addressed to design potential drug delivery systems for future viruses and different types of bacteria, for targeting patients with specifically identified genetic defects and developing them with a different sensitivity response in achieving targeted drug delivery through pH-responsive, light sensitivity, redox responsive, enzyme responsive, thermos responsive among others. Understanding the relationship between the drug delivery design, pharmacokinetics, and clinical effect is required for designing appropriate drug delivery systems for final clinical evaluations. A lot of research and clinical trials are still needed to foster the efficiency of these modern drug delivery systems.

In spite of the relevant issues mentioned above, the development of the first commercially available drug delivery system and advances in knowledge about in vivo results are important steps forward toward optimized design of more efficient formulations.

## Figures and Tables

**Figure 9:**
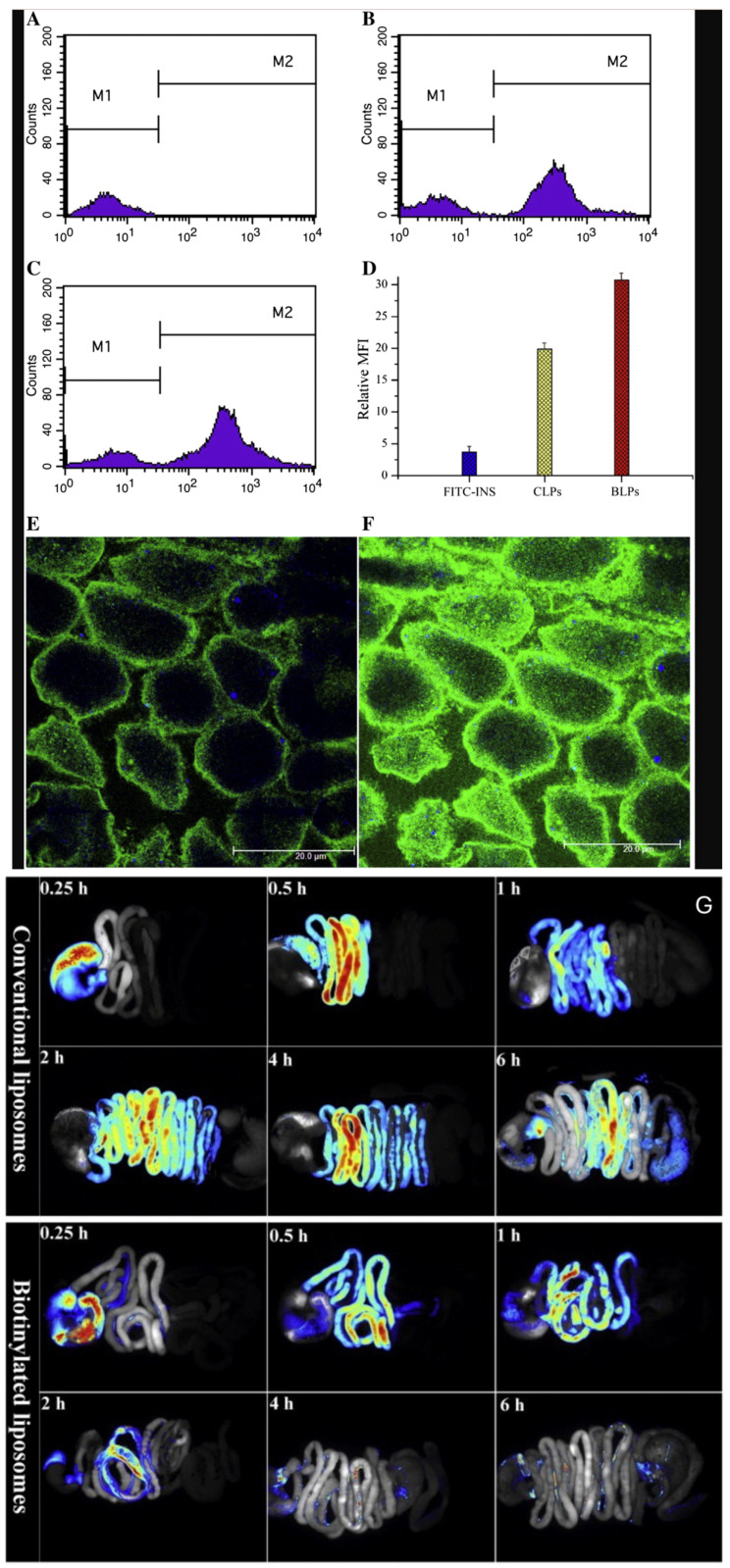
Flow cytometry of the uptake of FITC-INS at a concentration of 10 μg/mL in solution (**A**), Conventional liposomes (CLPs) (**B**), or (biotinylated liposomes) BLPs (**C**) and the mean fluorescence intensity (MFI) (**D**). Confocal microscopic images of Caco-2 cells incubated with fluorescence-labeled insulin-loaded CLPs (**E**) and BLPs (**F**). Ex vivo imaging of digestive tracts isolated immediately from rats following oral administration of CLPs and BLPs at different time points (**G**). Adopted from [76] with the permission from Elsevier.

**Table 1:** Effect of cyclodextrin (CD) on the solubility and stability of different drugs. Solubility enhancement is also shown for complexes of CD with drugs [17,18].

	Drugs	
	Enhanced Solubility	Enhanced Stability
β-CD	Valdecoxib (~3.5-fold), Diclofenac (~5-fold), Rofecoxib (~3-fold), Triamterene (~3-fold), Celecoxib (~5-fold), Benzocaine (~3-fold)	Glibenclamide, Diclofenac sodium, Quinaril, Flutamide
α-CD	3-hydroxyflavones (6-fold), Sidenafil (~1.6-fold)	---
δ-CD	Naftifine (~2-fold), Natamycin (~73-fold)	Paclitaxel, Spiranolactone, Digoxin
HP-β-CD	Bupivacaine (~1.5–4.5-fold), valsartan (18-fold), hesperetin (~10-fold), camptothecin (~30–50-fold), carprogen (~52-fold)	Rutin, Promethazine, Quinaril, Doxorubicin, Ganciclovir
DM-β-CD	Disoxaril (~3800-fold)	Promethazine
SBE-β-CD	Rofecoxib (~2-fold)	Paclitaxel, Melphalan and Carmustine

β-cyclodextrin (β-CD), α-cyclodextrin (α-CD), δ-cyclodextrin (δ-CD), 2-hydroxypropyl-β-cyclodextrin (HP-β-CD), 2,6-Di-O-methyl-β-cyclodextrin (DM-β-CD), sulfobutylether-β-cyclodextrin (SBE-β-CD).

**Table 2:** Available parenteral products containing CDs in Europe.

Drug	Brand Name	Administration Route	Bulk Weight (drug/CD)	Company
α-CD				
Alprostadil	Carveject^®^	Intracavitary	10 µg/324.7 mg	Pfizer, Inc.
HP-β-CD				
Diclofenac sodium	Dyloject^®^	Intravenous Intramuscular	75 mg/666 mg	Javelin Pharmaceuticals
Itraconazole	Sporanox™	Oral	10 mg/400 mg	Jansen
Televancin	Vibativ^®^	Intravenous	250 mg/2500 mg 750 mg/7500 mg	Astellas Pharma Theravance Biopharma
SBE-β-CD				
Aripiprazole	Ability^®^	Intramuscular	9.75 mg/195 mg	Bristol-Myers Squibb
Remdesivir	Veklury^®^	Intravenous	100 mg/3000 mg 100 mg/6000 mg	Gilead Sciences, Inc.
Voriconazole	Vfend^®^	Intravenous	200 mg/3200 mg	Pfizer, Inc.
Ziprasidone maleate	Geodon^®^ Zeldox^®^	Intramuscular	20 mg/294 mg	Pfizer, Inc.

**Table 3:** Examples of marketed CDs-based formulations for oral administration [24].

Drug	Brand Name	Dosage Form	Company
β-cyclodextrin (β-CD)			
Nimesulide	Nimedex^®^	Tablet	Novartis
Cetirizine	Zyrtec^®^	Chewing tablet	Losan Pharma/UCB Pharma
Ethynyl estradiol	Safyral/Beyaz/Lorina	Drospirenone; ethynyl estradiol; levomefolate calcium tablet	Bayer/Healthcare/Sandoz
Piroxicam	Cycladol/Brexin/Flamexin	Tablet	
2-hydroxypropyl-β-cyclodextrin (HP-β-CD)			
Perindopril tert-butylamine	Peridopril erbumine^®^	Tablet	Sandoz
Itraconazole	Sporanox^®^	Tablet	Jansen
β-cyclodextrin sulfobutyl ether (SBE-β-CD)			
Ziprazidone	Geodon	Capsule	Pfizer

**Table 4:** Examples of CDs-based formulations for ocular and nasal administration [29].

Drug	Brand Name	Dosage Form	Company
β-cyclodextrin (β-CD)			
Glucagon	Baqsimi™	Nasal spray	Eli, Lilly
2-hydroxypropyl-β-cyclodextrin (HP-β-CD)			
Indomethacin	Indocid	Eye drop solution	Chauvin
Randomly methylated-β-cyclodextrin (RM-β-CD)			
Cloramphenicol	Clorocil	Eye drop solution	Oftalder
17βEstradiol	Aerodiol	Nasal spray	Servier
2-hydroxypropyl-δ-cyclodextrin (HP-δ-CD)			
Diclofenac sodium salt	Voltaren	Eye drop solution	Novartis

**Table 5:** Marketed nanoemulsion as drug delivery systems.

Drug	Marketed Name	Indication	Manufactured
Vitamins A, D, E, K	Vitalipid^®^	Parenteral nutrition	Fresenius Kabi
Propofol	Diprivan^®^	Anestesic	Astra Zeneca
Diazepam	Diazemul^®^	Sedative	Actavis Nordic
Alprostadil palmitate	Liple^®^	Vasodilatador, platelet inhibitor	Mitsubishi Tanabe Pharma
Etomidate	Etomidat-Lipuro^®^	Anesthetic	Braun Melsungen
Dexamethasone-palmitate	Limethasone^®^	Rheumatoid arthritis	Mitsubishi Tanabe Pharma

**Table 6:** Some commercially available lipid base formulations for oral administration.

Drug	Trade Name	Type of Formulation	Excipients	Company
Cyclosporin A	Neoral^®^	Soft gelatin capsule	Corn oil-mono.-di-triglycerides, cremophor RH 40, di-α-tocopherol	Novartis
Ritonavir	Norvir^®^	Soft gelatin capsule	Oleic acid, Labrfil M-2125CS	Abbott
Valproic acid	Convulex^®^	Soft gelatin capsule	Diacetylated monoglycerides, cellulosic polymers, povidone, butylated hydroxyanisole	Pharmacia
Calcitriol	Rocaltrol^®^	Soft gelatin capsule	Fractionated triglyceride of coconut oil, parabens, sorbitol	Roche
Tipranavir	Aptivus^®^	Soft gelatin capsule	Polyoxyl 35 castor oil, propylene glycol, mono/diglycerides of caprylic/capric acid and gelatin	Boehringer Ingelheim

**Table 7:** Some marketed parenteral lipid emulsions for nutrition: oil nature and α-tocopherol content [45].

Product	Oil Source (% by Weight)	α-Tocopherol (µmol/L)	Company
Intralipid 20%	Soybean (100%)	87	Fresenius-Kabi; Germany
Lipofludin MCT	Coconut (50%) Soybean (50%)	502	B. Braun, Germany
Structolipid	Coconut (36%), Soybean (64%)	16	Fresenius-Kabi; Germany
ClinOleic	Olive (80%), Soybean (20%)	75	Baxter, France
Lipoplus	Coconut (50%), Soybean (40%) Fish (10%)	565	B. Braun, Germany
SMOFlipid	Coconut (30%), Soybean (30%), Olive (25%) Fish (15%)	500	Fresenius-Kabi; Germany
Omegaven	Fish (100%)	505	Fresenius-Kabi; Germany

**Table 8:** Representative marketed drug containing injectable emulsions.

Product	Active Drug	Composition	Company	Market
Vitalipid^®^	Vitamins A, D2, E, K1	Soy oil, egg Lecithin, Glycerol	Fresenius-Kabi; Germany	Europe
Stesolid^®^	Diazepam	Soy oil, acctylated monoglycerides, egg phospholipid, glycerol	Actavis, Ireland	Europe
Diazemuls^®^	Diazepam	Soy oil, acctylated monoglycerides, egg, glycerol, NaOH	Actavis, Ireland	Europe, Canada and Australia
Diprivan^®^	Propofol	Soy oil, egg lecithin, glicerol, disodium edelate, NaOH	Fresenius-Kabi; Germany	worldwide
Fluosol-DA^®^	Perfluorodecalin, Perfluorotripopylamine	Egg phospholipid, pluronic F68, potassium oleate, Glycerol	Green Cross and Alpha Therapeutics, Japan	worlwide

**Table 10:** Patents regarding oral liposomal and pro-liposome formulations.

Dosage Form	Formulation Composition	Drug	Outcome	Ref.
Hard capsule containing liposomes	Sorbitan Oleate: 2.0% Glutathione: 89.9% Purified Water: 4.0% Potassium sorbate: 0.2% Polysorbate 20: 2.0% Phospholipon 90 (DPPC): 2.0%	Glutathione	It was developed for the treatment of diseases associated with glutathione, such as Parkinson’s and cystic fibrosis. It is aimed to increase the effect of glutathione with the excipients in the formulation.	[83]
Soft capsule containing liposomes	Purified water: 10.0% Cyanocobalamin: 0.345% Cholesterol: 2.0% Vitamin E: 1.0% Benzyl alcohol: 1.0% Propylene glycol: 82.655%	* Vitamin E	The patent is specifically for active substances with low solubility. Formulations are prepared by injection method.	[84]
Soft capsule containing liposomes	Dextromethorphan: 5.5% DPPC: 2.0% Cholesterol: 0.2% PEG-12 glycery dioleate: 87.1% Purified water: 4.0% Potassium sorbate: 0.2% Vitamin E: 1.0%	** Dextromethorphan	When the prepared liposomal formulations contact with the aqueous medium, multilayered liposomes are formed. The liposomal formulation provides a prolonged release.	[85]
Tablet containing proliposomes	Glyburid: 27.56% DSPC: 52.12% Cholesterol: 13.5% DCP: 3.8%	Glyburid	With the cholesterol content in the formulation, it is aimed to reduce the particle size and increase the bioavailability by increasing the active substance transport. Different lipids were compared in the prepared proliposomal formulations and it was stated that DSPC was the most suitable lipid.	[86]

* This patent also includes Vitamin B12, Co-Enzyme Q10, and L-Carnitine. ** This patent also includes nifedipine and danazol.

## Data Availability

No new data were created or analyzed in this study.
